# Comparison of Immunomodulatory Therapies for Cardiovascular Clinical and Inflammatory Markers Outcomes in Mild to Moderately Ill Hospitalized Multisystem Inflammatory Syndrome in Children Patients

**DOI:** 10.3390/jcdd12090324

**Published:** 2025-08-25

**Authors:** Rashmitha Dachepally, Reem Sarkis, Alvaro DonaireGarcia, Meghana Kovvuri, Karunya Jayasimha, Adrija Chaturvedi, Amr Ali, Sirada Panupattanapong, Samir Latifi, Hemant Agarwal

**Affiliations:** 1 Department of Pediatric Critical Care Medicine, Children’s Nebraska, Omaha, NE 68114, USA; rdachepally@childrensnebraska.org; 2 Pediatric Emergency Medicine, Akron Children’s Hospital, Akron, OH 44302, USA; rsarkis@akronchildrens.org; 3 Department of Pediatric Critical Care, Driscoll Children’s Hospital Rio Grande Valley, Edinburg, TX 78539, USA; alvaro.garcia@dchstx.org; 4 Department of Pediatric Critical Care Medicine, Driscoll Children’s Hospital, Corpus Christi, TX 78411, USA; meghana.kovvuri@dchstx.org; 5 Department of Pediatric Cardiology, WVU Children’s, Morgantown, WV 26506, USA; karunyajayasimha@gmail.com; 6 Department of Pediatrics, Rainbow Babies & Children’s Hospital, Cleveland, OH 44106, USA; adrija.chaturvedi2@uhhospitals.org; 7 Department of Pediatric Cardiac Critical Care, WVU Children’s, Morgantown, WV 26506, USA; amr.ali1987@gmail.com; 8 Department of Pediatric Rheumatology, Cleveland Clinic Children’s, Cleveland, OH 44106, USA; panupas@ccf.org; 9 Department of Pediatric Critical Care, Cleveland Clinic Children’s, Cleveland, OH 44106, USA; latifis@ccf.org

**Keywords:** cardiac involvement, children, glucocorticoids, inflammatory biomarkers, intravenous immunoglobulin, MIS-C, treatment, ventricular function

## Abstract

Optimal treatment for non-critically ill multisystem inflammatory syndrome in children (MIS-C) remains unclear. We evaluated short-term outcomes in mild to moderately ill hospitalized MIS-C patients fulfilling CDC 2020 and CDC/CTSE 2023 criteria and treated between April 2020 and March 2022 with either intravenous immunoglobulin (IVIG) monotherapy (Group A, n = 17) or IVIG plus corticosteroids (GC) (Group B, n = 22). Cardiovascular clinical parameters, inflammatory markers, and cardiac imaging were compared on days 1, 3, and 5 relative to day 0. The two groups had no significant differences in demographics or illness severity. Group B showed improvement in heart rate (17.8; 95% CI [9.74, 25.8]), mean blood pressure (5.63 [1.61, 9.64]), and body temperature (1.45 [0.94, 1.95]) by day 1, followed by improvement in albumin (0.43 [0.2, 0.84]), CRP (7.56 [3.0, 12.11]), D-dimer (2344 [488.7, 4200.2]), ferritin (1448 [−609.4, 3505.5]), fibrinogen (110 [44.4, 176]), lymphocyte count (1006 [63.5, 1948]), and NT-proBNP (2901 [−349.3, 6153]) by day 3 and left ventricular ejection fraction by day 4–5 (3.84 [0.55, 8.23]). All results were statistically significant (*p* < 0.05). Group A required more additional therapies, with no difference in hospital stay. Our study concludes that combined IVIG and GC therapy yielded better short-term outcomes than IVIG monotherapy in this patient population, with improvement in cardiovascular clinical parameters preceding changes in inflammatory markers and cardiac imaging.

## 1. Introduction

Multisystem inflammatory syndrome in children (MIS-C) is the most serious pediatric disease associated with severe acute respiratory syndrome coronavirus 2 (SARS-CoV-2) [1]. Clinical features of organ dysfunction and hyperinflammation in MIS-C have comparable findings with Kawasaki disease, toxic shock syndrome, and macrophage activation syndrome [2,3,4,5,6,7,8]. The similarities of MIS-C with these disease processes and the absence of data from randomized controlled studies initially led to rapid application of intravenous immunoglobulin (IVIG) as the potential first-line treatment for MIS-C in clinical studies and recommended guidelines by national and international organizations, including the World Health Organization (WHO), the American College of Rheumatology (ACR), and the UK Royal College of Paediatrics and Child Health (RCPCH), with corticosteroids being reserved for patients unresponsive to IVIG therapy or more seriously ill patients [9,10,11]. Subsequently, large clinical studies and propensity-matched studies revealed conflicting results, with some reporting outcomes of patients, including cardiovascular dysfunction, intensive care unit stay, length of hospital stay, and need for additional medications, who received IVIG plus corticosteroids or monotherapy with IVIG or with corticosteroids as comparable, and other studies suggesting that the combination of IVIG plus corticosteroids is more efficacious [12,13,14,15,16,17]. A meta-analysis of the largest observational cohort studies revealed that combined treatment with IVIG and corticosteroids was associated with improved cardiovascular dysfunction compared to IVIG monotherapy, and treatment with corticosteroids alone was not associated with improved cardiovascular dysfunction compared to IVIG alone or IVIG and corticosteroids together [18]. The results of these observational studies, however, were hampered by risk of bias, challenges of treatment mixing in single-agent groups, heterogeneity of health-care access, and different national treatment guidelines. Very limited randomized controlled studies for the management of MIS-C have been undertaken, given the variability of presentation and clinical features of these patients [19,20]. One randomized study of MIS-C patients reported equivalent length of hospital stay, need for inotropes, duration of intensive care unit stay, major bleeding, and thrombotic events among patients receiving IVIG monotherapy or corticosteroids alone. The study, however, did not evaluate combined IVIG and corticosteroid management for these patients [19]. The other randomized open-label study evaluating IVIG monotherapy or high-dose corticosteroids or usual care revealed that steroids improved the duration of hospital stay compared to usual care, while IVIG provided no benefit [20]. There was, however, a high rate of off-protocol treatments administered to the ‘usual care’ groups, with 35% receiving IVIG and 50% receiving glucocorticoids in the two comparison groups, respectively. Recent guidelines by the American Academy of Pediatrics, ACR, and WHO recommend combined IVIG and GC therapy in the initial treatment of hospitalized patients, especially for moderately to severely ill MIS-C patients [21,22,23]. Conditional recommendations for the addition of GC to IVIG monotherapy have been suggested as substitute treatments for patients with mild illness who are unable to receive GC [21,23].

The clinical features of MIS-C vary from mild to severe manifestations [11,24,25,26,27,28]. The severity of MIS-C has steadily declined over the time course of the pandemic, with the frequency of pediatric intensive care unit (PICU) admission being halved from 55–60% for the Wuhan strain in 2020 to 20–25% for the Omicron strain in 2022 [12,13,14,15,16,21,29,30]. Cardiovascular dysfunction is the most frequently described physiological abnormality in MIS-C patients [31]. Myocardial involvement in MIS-C patients is common; however, it varies in clinical severity, and depressed left ventricular ejection fraction (LVEF) does not always result in the use of vasopressors [11,13]. Limited studies have evaluated cardiovascular clinical parameters or inflammatory marker responses of different therapies in mild to moderately ill hospitalized MIS-C patients [17,18,27,31,32]. The criteria to define MIS-C have also undergone modifications as the MIS-C pandemic has evolved over the years [5,6,7,8]. Initially in 2020, at the beginning of the pandemic, multiple organizations, including the Centers for Disease Control and Prevention (CDC), RCPCH, and WHO, defined MIS-C on the basis of the presence of fever, multiorgan involvement, elevated inflammatory markers, evidence of current or recent SARS-CoV-2 infection or exposure, and exclusion of other potential diagnoses [5,6,7]. Subsequently, in 2023, the Council of State and Territorial Epidemiologists (CSTE) and CDC released a new MIS-C case definition following an analysis of the diagnostic performance of the previous criteria and comparing MIS-C with other hyperinflammatory syndromes [8]. The primary aim of our study was to assess changes in cardiovascular clinical parameters, inflammatory markers, and cardiac imaging during hospital stay in mildly to moderately ill hospitalized MIS-C children diagnosed by the CDC 2020 and the CDC/CSTE 2023 definitions receiving IVIG monotherapy versus combined IVIG and GC therapy [6,8]. Our secondary aim was to assess the need for additional medications, transfer to the PICU, and length of hospital stay in these patients.

## 2. Materials and Methods

A single-center, retrospective, observational chart review was undertaken at a tertiary children’s hospital. The Cleveland Clinic Foundation Institutional Review Board approved the study and granted a waiver of consent (IRB # 22-170). All children < 21 years of age admitted to the hospital with a primary diagnosis of MIS-C per CDC 2020 and CDC/CTSE 2023 criteria between April 2020 and March 2022 were evaluated [6,8]. The MIS-C patients were categorized as mild, moderate, or severe as previously reported: (a) mild: no vasoactive requirement, minimal respiratory support and/or minimal signs of organ injury, normal ventricular function (LVEF > 55%); (b) moderate: Vasoactive-Inotrope Score (VIS) ≤ 10, significant supplemental oxygen requirement, and/or mild or isolated organ injury, mild to moderate left ventricular dysfunction (LVEF: 30–55%); and (c) severe: VIS > 10, invasive ventilatory support and/or moderate or severe organ injury, severe left ventricular dysfunction (LVEF < 30%) [22,23,24,26,32]. Exclusion criteria were patients not fulfilling both CDC 2020 and CDC/CTSE 2023 criteria, patients treated at other institutions prior to arrival, or patients having severe MIS-C illness.

De-identified longitudinal data including patient demographics, presence of comorbidities, clinical features at presentation, organ systems involved, clinical parameters, laboratory studies of inflammatory markers, cardiac and radiographic imaging, immunomodulatory therapies, supportive and antithrombotic treatments, and hospital course were collected retrospectively from electronic health records and stored in a secure database [33]. History of contact with a person with suspected or confirmed COVID-19 or laboratory-confirmed SARS-CoV-2 infection (positive for SARS-CoV-2 real-time reverse-transcriptase-polymerase-chain-reaction or antibody test during hospitalization) for all patients was recorded. Immunomodulatory therapies, including IVIG, GC, and biologics like interleukin-1–receptor antagonist, tumor necrosis factor α inhibitor, or interleukin-6–receptor antagonist given any time during the hospital course were recorded. 

The first calendar day of immunomodulatory treatment was defined as day 0, and subsequent treatment and outcomes were defined relative to this definition [13,15]. IVIG monotherapy or IVIG and GC given within 24 h of one another on day 0 were considered as primary treatment [13,17]. Any different immunomodulatory therapy administered 24 h after the primary treatment was considered additional medications/secondary treatment [13,34]. Time was measured in calendar days from treatment initiation [13]. Days 1, 3, and 5 were the first, third, and fifth calendar days after day 0 [13,35]. Cardiovascular clinical parameters, including heart rate, blood pressure, and body temperature, were recorded daily. All MIS-C patients had initial laboratory tests performed on admission and an echocardiographic examination on day 0. Echocardiogram studies were evaluated for LVEF, coronary artery dilation, and presence of pericarditis or pericardial effusion. LVEF was based on modified Simpson’s method and categorized as either normal (≥55%), mild (45–54%), moderate (30–44%), or severe impairment (<30%) [36]. Dilation of the coronary artery with a z score of ≥2.5 or any aneurysm was recorded [37]. Subsequent investigations and treatments were at the discretion of the multidisciplinary team based on the response to immunomodulatory therapy. Repeat echocardiographic examination was generally undertaken on day 4–5 of therapy. 

Patients were divided into two groups based on the immunomodulatory therapy they received on day 0. Patients who received IVIG monotherapy on day 0 (Group A) were compared to patients who received combined (IVIG and GC) therapy on day 0 (Group B). Patients were assessed for changes in clinical parameters, including delta decrease in heart rate, delta increase in mean blood pressure, delta decrease in body temperature, and delta changes in commonly tested inflammatory markers, including albumin, C-reactive protein (CRP), D-dimer, ferritin, fibrinogen, lymphocyte count, neutrophil count, N-Terminal pro-brain natriuretic peptide (NT-proBNP), and LVEF for primary outcomes. Delta was defined as the difference between the measured clinical parameter or inflammatory marker level between day 0 and day 1 of treatment, day 0 and day 3 of treatment and day 0 and day 5 of treatment. Patients were assessed for additional immunomodulatory therapy and hospital course, including the need for PICU admission and length of hospital stay for secondary outcomes. All laboratory testing was undertaken at the primary institution’s central laboratory. Serum albumin levels were analyzed by bromocresol green. CRP and D-dimer levels were analyzed using turbidimetric immunoassay. Serum ferritin and NT-proBNP levels were analyzed by electro chemiluminescence immunoassay. Fibrinogen levels were analyzed by automated optical clot detection. IL-6 levels were measured by immunoenzymatic assay. Complete blood count and differential cell count, including absolute neutrophil count and absolute lymphocyte count, were measured by automated cell counter. 

Data was described using medians and ranges or means and standard deviations for continuous variables and counts and percentages for categorical variables. Treatment groups were compared on baseline demographic and clinical characteristics and on outcomes using two-sample *t*-tests or nonparametric Wilcoxon rank sum tests for continuous and ordinal characteristics, and chi-square or Fisher’s exact tests for categorical characteristics. All analyses were performed on a complete-case basis. All tests were two-tailed and performed at a significance level of 0.05. SAS 9.4 software (SAS Institute, Cary, NC, USA) was used for all analyses.

## 3. Results

A total of 39 MIS-C patients were included in our study. Nineteen patients were excluded from the study, as 5 patients did not fulfill the CDC/CTSE 2023 definition, 2 patients had received prior treatment, and 12 patients had severe illness. Seventeen patients received IVIG monotherapy on day 0 (Group A), and 22 patients received a combination of IVIG and GC therapy on day 0 (Group B). 

The demographic details, presence of comorbidities, presence of SARS-CoV-2 infection, organ systems involvement per the CDC 2020 and CDC/CTSE 2023 definitions, and severity of illness are shown in Table 1. There was no difference in the demographic details between the two groups, with 38% of patients having obesity and 33% having chronic medical illnesses. Per the CDC 2020 criteria, an average of 4 organ systems were involved in our study population, with gastrointestinal, mucocutaneous, and cardiovascular systems being the most commonly involved systems. Per the CDC/CTSE 2023 criteria, an average of 3 organ systems were involved, with gastrointestinal, hematological, and mucocutaneous systems being the most commonly involved systems. The cardiovascular system was involved in 54% of the patients, and shock as a separate criterion was present in 26% of our study population. Mild severity of illness was present in 54% of patients, and moderate severity of illness was present in 46% of patients, with no significant differences between the two groups.

The clinical features and presenting signs and symptoms of MIS-C per the CDC/CTSE 2023 definition are shown in Table 2. All patients had fever with an average duration of fever and illness for 5 days. Tachycardia was present in 97% of our study population. Hypotension was present in 15% and shock was present in 26% of the patients, whereas depressed LVEF was present in 38% of our patient population. Abdominal pain and vomiting were the most common gastrointestinal manifestations, whereas low lymphocyte count was the most prevalent hematological manifestation in our patient population.

Initial laboratory testing, including inflammatory marker levels, echocardiography, and chest X-ray for the two groups, is shown in Table 3. An average of 6 inflammatory markers were positive in our study population, with elevated CRP and IL-6 levels being present in 100% of the patients tested and elevated fibrinogen, low lymphocyte count, and elevated NT-proBNP level being present in more than 85% of the patients tested. Pericarditis was the most common echocardiographic abnormality seen in our patients, with only 7.6% of patients revealing coronary artery aneurysm. Electrocardiograms revealed no arrhythmia other than sinus tachycardia in our patients. The liver function tests, kidney function tests, urinalysis at admission, and chest X-ray results for our cohort of mild to moderately ill MIS-C patients are shown in Supplementary Table S1. There were no significantly abnormal test results at baseline in these organ systems, and there were no differences between the patients in the 2 groups.

Management of MIS-C patients, including immunotherapeutic medications, supportive care, and antithrombotic therapy administration, is shown in Table 4. There was minimal delay in the initiation of therapy following admission to the hospital in our patient population. Of the 16 patients receiving fluid resuscitation, 10 patients received it for management of shock and 6 patients for hypotension or clinical signs of dehydration. Of the 10 patients with shock, 3 (1 in Group A and 2 in Group B) patients were managed with 45 ± 14.7 mL/kg fluid bolus resuscitation without inotrope support, and 7 (1 in Group A and 6 in Group B) patients were managed with 30 ± 14.3 mL/kg fluid bolus resuscitation and inotrope support. All patients received a dose of 2 g/kg of IVIG for their treatment. The majority of our patients received low-dose corticosteroid therapy in divided doses for their treatment. Six of eight patients received high-dose pulse steroids for moderately depressed LVEF and hypotension, 1 for acute kidney injury, and 1 for neuroirritability. Four of the 7 Group A patients received GC in addition to IVIG therapy for persistent fevers and worsening inflammatory markers, 2 patients for persistent fevers and low LVEF findings, and 1 patient for persistent fevers and hypotension. IL-1 inhibitor therapy was added to 7 patients: 2 in Group A and 5 in Group B for severely depressed LVEF and persistently elevated inflammatory markers. The majority of our patients received antiplatelet therapy, and Group B patients received significantly more anticoagulation therapy as compared to Group A patients. Anticoagulation therapy was administered to patients with moderate to severely depressed LVEF and markedly elevated D-dimer and fibrinogen levels. Deep vein thrombosis was not detected in any patient that was evaluated for suspected thrombosis.

**Figure 1:**
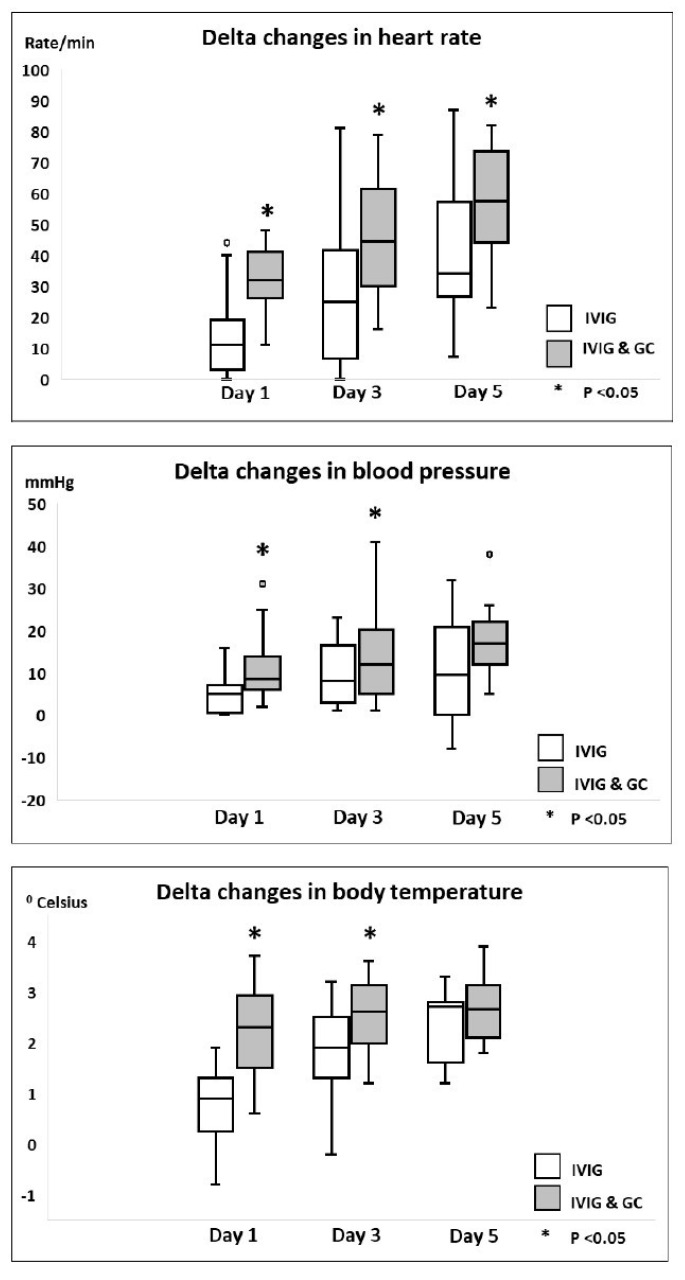
Comparison of delta changes in clinical parameters in mild to moderately ill hospitalized MIS-C patients receiving IVIG monotherapy versus combined IVIG and GC therapy.

Cardiovascular clinical parameters during hospitalization, including delta changes in heart rate, mean blood pressure, and temperature on day 1, day 3, and day 5 as compared to day 0 of therapy and the hospital course for both groups, are shown in Table 5 and Figure 1. Patients in Group B had significant delta changes in all clinical parameters, including heart rate, mean blood pressure, and temperature, on day 1 and day 3 following initiation of therapy as compared to Group A patients. There was significant earlier resolution of fever in Group B patients as compared to Group A patients. 18 patients (6 in Group A and 12 in Group B) were admitted to the PICU. 10 patients were transferred to the PICU after initiation of therapy for escalation of care. The time interval for transfer from the regular pediatric ward to the PICU was 1 [1,2] day. The hospital length of stay was not significantly different between the two groups.

**Figure 2:**
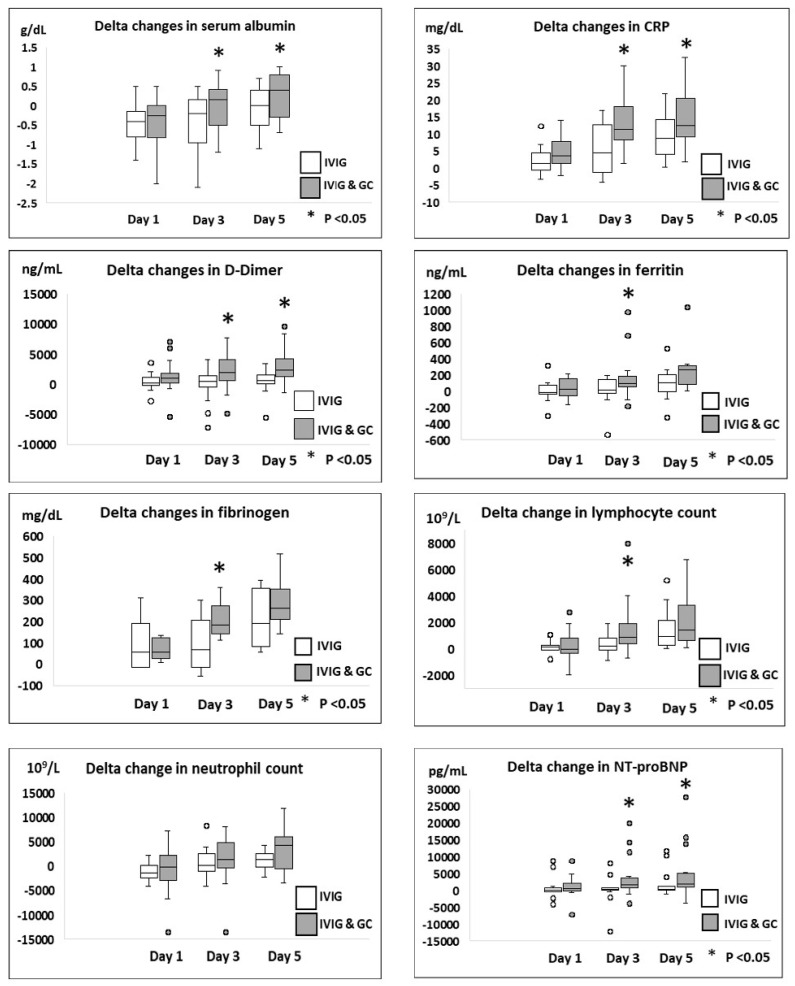
Comparison of delta changes in inflammatory markers in mild to moderately ill hospitalized MIS-C patients receiving IVIG monotherapy versus combined IVIG and GC therapy.

**Figure 3:**
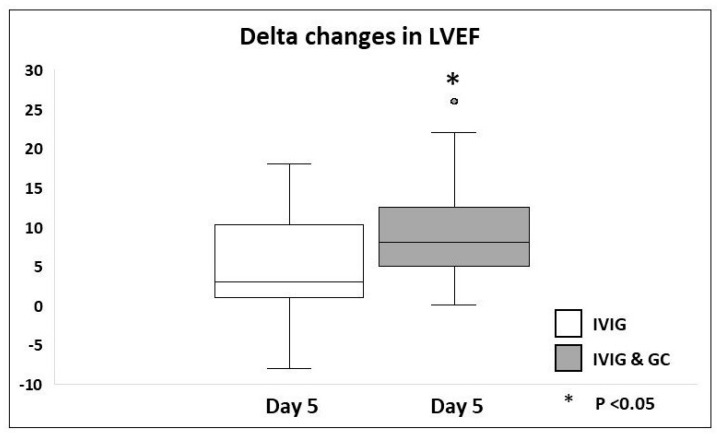
Comparison of delta changes in left ventricular ejection fraction (LVEF) in mild to moderately ill hospitalized MIS-C patients receiving IVIG monotherapy versus combined IVIG and GC therapy.

Laboratory studies and cardiac imaging studies, including delta changes in levels of inflammatory markers and LVEF on day 1, day 3, and day 5 as compared to day 0 for both groups, are shown in Table 6, Figure 2 and Figure 3. There were no significant delta changes in any of the inflammatory markers tested on day 1 following initiation of therapy as compared to day 0. However, thereafter, a significant improvement in delta changes of 7 of the 8 inflammatory markers, including serum albumin, CRP, D-dimer, ferritin, fibrinogen, lymphocyte count, and NT-proBNP level, was seen on day 3 as compared to day 0 in group B patients versus group A patients. Delta changes in serum albumin, CRP, and D-dimer levels continued to show significant improvement on day 5 as compared to day 0 in Group B patients as compared to Group A patients. A significant delta improvement in LVEF% was seen on day 4–5 of therapy in Group B patients as compared to Group A patients. All patients had LVEF ≥ 55% on day 4–5 of therapy.

## 4. Discussion

Our study results reveal that short-term outcomes are significantly better in mild to moderately ill hospitalized MIS-C patients receiving combined IVIG and GC immunomodulating therapy as compared to IVIG monotherapy. Improvements in cardiovascular clinical parameters were observed earlier than changes in inflammatory markers and cardiac imaging among patients receiving combined therapy. Patients treated with IVIG monotherapy required more secondary immunomodulators, although there was no significant difference in the length of hospital stay between the two groups.

The time from onset of symptoms to hospital admission, clinical features on presentation, and number of organ systems involved in our study patients were similar to previous reports [1,12,13,14,16,24,30,38,39,40,41,42,43,44,45]. Cardiovascular clinical parameters, including heart rate, mean blood pressure, and percentage of tachycardic patients on admission, were also similar to previous studies [24,25,30,45,46]. We elected to assess the temporal resolution of tachycardia and blood pressure changes in our study, as cardiovascular dysfunction is the most prevalent physiological abnormality in MIS-C patients, and hypotension, shock, and vasopressor support were significantly less in our study patients. Fifteen percent of our patients developed hypotension compared to 36–61% in previous studies, 26% of our patients developed shock compared to 40–65% in previous studies, and 17% of patients required vasopressor support compared to 31–64% in previous studies [12,13,16,20,27,30,35,39,40,42,45,47,48,49,50]. Assessment of clinical parameters in our study revealed significant delta changes in heart rate, mean blood pressure, and temperature within 1 day in patients receiving combined IVIG and GC therapy as compared to IVIG monotherapy. A single-center study of 22 MIS-C patients has reported significant improvement in heart rate and blood pressure within 24 h of initiating GC therapy as compared to IVIG therapy [24]. A meta-analysis of three large propensity-matched studies of severely ill MIS-C patients reported combined IVIG and GC therapy to be associated with less persistent cardiovascular dysfunction, defined as the need for inotrope support or LVEF < 55% on day 2 of therapy, compared to patients receiving IVIG monotherapy [17]. A significant earlier resolution of fever in our patients receiving combined IVIG and GC compared to IVIG monotherapy has been previously reported [17,18,35]. A rising trend of additional immunomodulatory therapy after 1 day of initiation of treatment was seen in 54% of MIS-C patients receiving IVIG therapy compared to 24% in patients receiving combined IVIG and GC therapy in our study as previously reported [16,17,18,35,51]. A lower number of patients required transfer to the PICU from the floor in our study than the previously reported rate of 30–33%, likely due to the exclusion of severely ill patients in our study [51,52,53]. A lack of significant difference in length of hospital stay was seen in our patients receiving combined IVIG and GC therapy versus IVIG monotherapy. There have been mixed findings on the association of length of hospital stay with different immunomodulatory therapies in literature [17,20,35,50].

The positivity rate for inflammatory markers in our study of albumin (61%), CRP (100%), D-dimer (64%), ferritin (58%), fibrinogen (87%), and NT-proBNP (89%) with an elevation of at least 5 of the 8 inflammatory markers tested was similar to previous reports [1,22,27,54,55]. Our study, however, did not reveal a significant difference in the levels of inflammatory mediators in patients receiving IVIG monotherapy versus combined IVIG and GC therapy as described previously [22,25,30,52,56,57,58]. A lack of inclusion of severely ill MIS-C patients in our study may have been responsible, as MIS-C is a hyperinflammatory syndrome with levels of albumin, CRP, D-dimer, ferritin, fibrinogen, lymphocyte count, and NT-pro-BNP being directly or indirectly affected by inflammatory mediators [59,60,61,62,63,64]. Serial assessment of changes in the levels of inflammatory markers during the hospital stay in our study revealed significant changes in albumin, CRP, D-Dimer, ferritin, fibrinogen, and lymphocyte count from the third day of combined IVIG and GC therapy compared to IVIG monotherapy. These findings corroborate previous studies of severely ill MIS-C patients receiving combined IVIG and GC therapy that reported albumin, CRP, D-dimer, ferritin, lymphocyte count levels halved from their peak value or reached near-normal levels within 3 to 5 days of initiation of treatment [31,65]. A significant change in NT-proBNP levels on day 3 and day 5 of combined IVIG and GC therapy versus IVIG monotherapy was observed in our study, similar to previous studies [41,66,67]. Our study patients receiving IVIG monotherapy demonstrated an elevation of NT-proBNP levels on day 1 of IVIG monotherapy with a gradual decrease over the next 5 days as previously reported [48]. Exposure to increased volume overload and viscosity of IVIG infusion to an inflamed and edematous myocardial tissue may be responsible for the increased filling pressures and release of NT-proBNP [68,69]. Likewise, daily echocardiography studies of MIS-C patients in a previous study have reported the lowest LVEF to be detected at 1–2 days following admission to the hospital [45]. Thereafter, significant changes in LVEF on day 4 to 5 of combined IVIG and GC therapy seen in our patients were similar to previous reports of improvement by day 3 to day 4 of combined therapy with complete recovery by day 5 to day 7 of therapy [26,41,45,69]. Improvement in NT-proBNP closely followed improvement in LVEF as previously reported [41].

The goals of MIS-C therapy are to stabilize the patients with multiorgan dysfunction, reverse the hyperinflammatory response, and prevent long-term sequelae [9]. Several guidelines for MIS-C treatment have been published, but no consensus has been reached [6,9,11,34,70]. The pathogenetic mechanism of MIS-C has not been fully elucidated in the literature. It is presumed to be a post-infectious disorder [71,72]. The subsequent immunological activation in MIS-C shows features of autoimmunity or dysregulated immune response as demonstrated by the presence of autoantibodies targeting cardiac tissue, the gastrointestinal tract, endothelial and immune cells, and hyperinflammation as demonstrated by markedly elevated concentrations of a broad spectrum of serum cytokines, including IL-1β, IL-6, IL-8, IL-10, IL-17, IL-18, IFN-γ, and TNF, leading to elevated inflammatory markers and multi-organ dysfunction [59,73,74,75,76]. IL-6 levels were markedly elevated in our patients. Our study results reveal that there may be a role for combined therapy of IVIG and GC in the management of mild to moderately ill hospitalized MIS-C patients. Hypotension in MIS-C patients results from myocardial dysfunction or inflammation-associated vasodilation and capillary leakage [77]. The initial improvement in cardiovascular clinical parameters, including heart rate and blood pressure, in our study may be related to the nongenomic effects of GC on immune and endothelial cells, including membrane permeability, adenosine triphosphate production, and T-cell receptor signaling that cause inhibition of vasodilatation and decreased vascular permeability soon after administration of GC [78,79,80]. Subsequent response of the combined therapy in MIS-C may be related to GC and IVIG affecting different pathways. Systemic inflammation, autoantibodies, and cytokine storm contribute to cardiac pathology in MIS-C [81]. Glucocorticoids may be acting genomically in MIS-C by inhibiting nuclear transcription of proinflammatory cells in the signaling pathway, resulting in reduced release of a diverse array of proinflammatory cytokines, including IL-1, TNF-α, IL-6, IL-11, and IL-16, causing inhibition of inflammatory mediator production and targeting nonspecific systemic inflammation [59,60,61,62,63,79,80,82]. Likewise, IVIG may be acting by inhibiting autoantibody production or directly neutralizing autoantibodies to accelerate the clearance of autoantibodies in MIS-C patients, similar to other illnesses, causing limitation of tissue injury and organ dysfunction with sustained improvement in cardiovascular parameters, LVEF, and inflammatory mediators [83,84,85,86].

Our study has certain limitations that should be considered. First, our study is a single-center, retrospective design without randomization, which may impact the generalizability and applicability of our findings across different clinical settings. Second, we did not perform propensity score matching due to the small sample size and relative similarity between groups. Third, the absence of standardized severity criteria for MIS-C in the literature poses challenges for comparing our results with those of other studies and for uniformly classifying clinical conditions [5,24,25,27,58,87]. Fourth, we did not follow patients’ symptoms and signs, such as abdominal pain, vomiting, diarrhea, skin rash, conjunctivitis, and inflammation of oral mucosa, during their time period of hospitalization to report differences between the two groups. Fifth, we did not study serial IL-6 levels as one of the elevated inflammatory markers in our cohort of mild to moderately MIS-C patients during their short course of their hospitalization. Elevated IL-6 levels on admission in our study population were similar to previously reported studies [29,88,89]. A prognostic value of elevated IL-6 levels in MIS-C patients at admission to predict development of cardiovascular dysfunction and prolonged length of stay has been reported in literature without their serial measurements [90,91]. Sixth, decision making regarding laboratory testing, imaging, escalation of care, and suitability for discharge was at the discretion of the relevant responsible medical teams. Seventh, our study protocol did not incorporate steroid monotherapy for management of mildly to moderately ill MIS-C patients. Observational studies and randomized clinical trials have reported similar outcomes between MIS-C patients receiving GC versus IVIG therapy versus combined therapy [15,16,17,19,20,27]. MIS-C and Kawasaki disease, however, have overlapping clinical features with misdiagnosis in approximately one-fifth of patients [92]. There is concern that withholding IVIG therapy from Kawasaki patients misdiagnosed with MIS-C may have detrimental effects on coronary dilatations and aneurysms in Kawasaki disease [78]. Eighth, our study evaluated only short-term outcomes. Long-term follow-up is planned and would be beneficial to define the late outcomes for these patients. Lastly, the study’s timeframe, which spans two years, coincides with an evolving landscape of MIS-C management, reflecting changes in clinical practices that could influence the observed outcomes [9,21,29,30,71,93,94,95].

## 5. Conclusions

Our study results reveal a beneficial role of combined IVIG and GC therapy versus IVIG monotherapy for short-term outcomes of mildly to moderately ill hospitalized MIS-C patients. The combined IVIG and GC therapy in these patients facilitates an earlier improvement in cardiovascular parameters than inflammatory marker levels and cardiac imaging, highlighting the potential for this treatment strategy to offer a dual benefit in managing the disease’s immediate cardiovascular symptoms and underlying inflammation. The inherent limitations of our study suggest the need for future randomized controlled trials to validate these findings and refine treatment protocols for MIS-C.

## Figures and Tables

**Table 1:** Demographics, epidemiology, clinical definition and severity of illness of hospitalized MIS-C patients treated with IVIG monotherapy versus combined IVIG and corticosteroid therapy.

Characteristic	All Patients (n = 39)	IVIG Treatment (n = 17)	IVIG and Corticosteroid Treatment (n = 22)	*p* Value
**Demographics**				
Age (years)	7.6 ± 4.6	7.9 ± 4.6	7.3 ± 4.7	0.70 *
Weight (kilograms)	30 [18, 52]	32 [17, 52]	29 [21, 46]	0.98 ^†^
Gender: Male	25 (64)	12 (71)	13 (59)	0.46 ^‡^
Race and ethnicity				0.84 ^§^
Asian	3 (7.7)	1 (5.9)	2 (9.1)	
Black	13 (33)	7 (41)	6 (27)	
Hispanic or Latino	4 (10)	2 (12)	2 (9.1)	
White	19 (49)	7 (41)	12 (55)	
**Presence of comorbidities**				0.61 ^§^
Obesity	15 (38)	7 (41)	8 (36)	0.76 ^‡^
Chronic medical illness ^a^	13 (33)	4 (24)	9 (41)	0.25 ^‡^
**Presence of SARS-CoV-2 infection**		17 (100)	22 (100)	
Positive SARS-CoV-2 NAAT or antigen	4 (10)	1 (6)	3 (17)	0.79 ^§^
Positive SARS-CoV-2 antibody	10 (26)	3 (18)	7 (32)	0.52 ^§^
Exposure to suspected or confirmed COVID-19 case	18 (46)	13 (76)	15 (68)	0.83 ^§^
**Organ system involvement per 2020 CDC MIS-C definition** [6]				
Cardiac	32 (82)	11 (65)	21 (95)	0.04 ^§^
Hematologic	27 (69)	11 (65)	16 (73)	0.59 ^§^
Gastrointestinal	37 (95)	17 (100)	20 (91)	0.59 ^§^
Mucocutaneous	30 (77)	12 (71)	18 (82)	0.46 ^§^
Neurologic	14 (36)	5 (29)	9 (41)	0.46 ^§^
Musculoskeletal	8 (21)	3 (18)	5 (23)	0.99 ^§^
Renal	1 (2.6)	0 (0)	1 (4.5)	0.99 ^§^
Respiratory	9 (23)	1 (5.9)	8 (36)	0.052 ^§^
Number of organ systems involved	4 [3, 5]	4 [3, 4]	4 [4, 5]	0.025 ^†^
**Multisystem involvement per 2023 CSTE/CDC MIS-C surveillance definition** [8]				
Cardiac	21 (54)	7 (41)	14 (64)	0.28 ^§^
Hematologic	34 (87)	15/17 (88)	19/22 (90)	0.99 ^§^
Gastrointestinal	37 (95)	17 (100)	20 (91)	0.59 ^§^
Mucocutaneous	30 (78)	12 (71)	18 (82)	0.46 ^§^
Number of organ systems involved	3 [2, 4]	3 [2, 4]	3 [2, 4]	0.28 ^§^
Shock	10 (26)	2 (12)	8 (36)	0.16 ^‡^
**Severity of illness**				0.23 ^‡^
Mild	21 (54)	11 (65)	10 (45)	
Moderate	18 (46)	6 (35)	12 (55)	

Data presented as mean ± SD, median [P5%, 75%], N (column %), *: Satterthwaite *t*-test, ^†^: Wilcoxon rank sum test, ^‡^: Pearson’s chi-square test, ^§^: Fisher’s exact test, ^a^: h/o allergic rhinitis or eczema, asthma, congenital heart disease, hematological diseases, immunological diseases.

**Table 2:** Clinical features of hospitalized MIS-C patients treated with IVIG monotherapy versus combined IVIG and corticosteroid therapy.

Characteristic	All Patients (n = 39)	IVIG Treatment (n = 17)	IVIG and Corticosteroid Treatment (n = 22)	*p* Value
**Clinical features**				
Duration of illness (days)	5.0 [4.0, 6.0]	6.0 [5.0, 6.0]	5.0 [4.0, 5.0]	0.17 ^†^
Duration of fever (days)	5.0 [4.0, 6.0]	5.0 [4.0, 6.0]	5.0 [4.0, 5.0]	0.59 ^†^
**Clinical symptoms**				
Presence of fever	39 (100)	17 (100)	22 (100)	
**Clinical signs**				
Heart rate (rate/min)	129 [103, 139]	116 [101, 139]	137 [115, 137]	0.15 ^†^
Tachycardic patients	38 (97)	16 (94)	22 (100)	0.44 ^§^
Mean arterial blood pressure (mmHg)	65 [54, 72]	67 [57, 72]	61 [54, 73]	0.81 ^†^
Hypotension	6 (15)	2 (12)	4 (18)	0.91 ^‡^
Shock	10 (26)	2 (12)	8 (36)	0.16 ^‡^
**Clinical symptoms and findings per 2023 CSTE/CDC MIS-C surveillance definition** [8]				
Cardiac	21 (54)	7 (41)	14 (64)	0.28 ^§^
LVEF < 55% in patients	15 (38)	5 (29)	10 (45)	0.30 ^§^
Coronary artery dilatation	3 (8)	1 (6.7)	2 (9.1)	0.99 ^§^
Elevated troponin levels	5 (13)	1 (6.7)	4 (18)	0.43 ^§^
Gastrointestinal	37 (95)	17 (100)	20 (91)	0.59 ^§^
Abdominal pain	33 (85)	14 (82)	19 (86)	0.92 ^§^
Vomiting	34 (87)	16 (94)	18 (81)	0.51 ^§^
Diarrhea	22 (56)	7 (41)	15 (68)	0.17 ^§^
Hematological	34 (87)	15/17 (88)	19/22 (90)	0.99 ^§^
Absolute Lymphocyte count < 1000/mcL	34 (87)	15/17 (88)	19/22 (90)	0.99 ^§^
Platelet count < 150,000/mcL	7 (18)	2/17 (12)	5/22 (23)	0.38 ^§^
Mucocutaneous	30 (77)	12 (71)	18 (82)	0.46 ^§^
Skin rash	22 (56)	10 (59)	12 (57)	0.92 ^§^
Inflammation of oral mucosa	18 (46)	8 (47)	10 (45)	0.82 ^§^
Conjunctivitis	18 (46)	9 (53)	9 (41)	0.45 ^§^
Edema/peeling of peripheral extremities	13 (33)	4 (24)	9 (41)	0.42 ^§^

Data presented as median [P5%, 75%], N (column %), ^†^: Wilcoxon rank sum test, ^‡^: Pearson’s chi-square test, ^§^: Fisher’s exact test, LVEF: left ventricular ejection fraction; mcL: microliter

**Table 3:** Inflammatory markers, echocardiographic and radiographic results of hospitalized MIS-C patients treated with IVIG monotherapy versus combined IVIG and corticosteroid therapy.

Characteristic	All Patients (n = 39)	IVIG Treatment (n = 17)	IVIG and Corticosteroid Treatment (n = 22)	*p* Value
**Inflammatory markers**				
Number of inflammatory markers tested	8 [7, 9]	8 [7.7, 8]	8 [7, 9]	0.98 ^†^
Number of positive inflammatory markers	6 [5, 8]	5 [4, 7.5]	7 [5, 8]	0.23 ^†^
Albumin level ≤ 3 g/dL ^††^	24/39 (61)	11/17 (65)	13/22 (59)	0.72 ^‡^
Albumin level (g/dL)	2.8 [2.3, 3.2]	2.8 [2.65, 3.1]	2.8 [2.2, 3.2]	0.58 ^†^
C-reactive protein levels ≥ 3/dL ^††^	39/39 (100)	17/17 (100)	22/22 (100)	
C-reactive protein levels (mg/dL)	13.5 [10.2, 19.7]	13.5 [8.05, 11.1]	14.2 [10.6, 21.5]	0.39 ^†^
D-dimer levels > 3000 ng/mL ^††^	25/39 (64)	8/17 (47)	17/22 (77)	0.051 ^‡^
D-dimer levels (ng/mL)	4110 [2050, 7390]	2700 [2050, 5270]	4505 [3570, 7390]	0.32 ^†^
Ferritin levels > 500 ng/mL ^††^	22/38 (58)	8/17 (47)	14/21 (67)	0.22 ^‡^
Ferritin levels (ng/mL)	569 [318, 1018]	494 [228, 663]	576 [433, 1050]	0.11 ^†^
Fibrinogen levels > 400 mg/dL ^††^	26/30 (87)	11/13 (85)	15/17 (88)	0.99 ^§^
Fibrinogen levels (mg/dL)	552 [450, 638]	577 [459, 744]	486 [430, 609]	0.17 *
Interleukin-6 levels > 7 pg/mL ^††^	23/23 (100)	11/11 (100)	12/12 (100)	
Interleukin-6 levels (pg/mL)	173 [42, 411]	57 [31, 318]	196 [139, 675]	0.074 ^†^
Lymphocyte count < 1 × 10^9^/L ^††^	34/39 (87)	15/17 (88)	19/22 (90)	0.99 ^§^
Lymphocyte count (×10^9^/L)	0.84 [0.63, 1.06]	0.89 [0.68, 1.06]	0.74 [0.60, 0.96]	0.36 ^†^
Neutrophil count > 7.7 × 10^9^/L ^††^	28/39 (72)	10/17 (59)	18/22 (82)	0.16 ^§^
Neutrophil count (×10^9^/L)	10.16 [7.32, 14.29]	9.54 [4.4, 11.29]	10.63 [9.21, 15.48]	0.046 ^†^
NT-proBNP level > 400 pg/mL	34/38 (89)	12/16 (75)	22/22 (100)	0.024 ^§^
NT-proBNP levels (pg/mL)	2102 [760, 5762]	792 [437, 6132]	2986 [1084, 5741]	0.047 ^†^
**Echocardiographic results**				
LVEF < 55% in patients	15 (38)	5 (29)	10 (45)	0.30 ^‡^
LVEF on admission	57 [49, 64]	63 [49, 66]	58.5 [49, 61]	0.13 ^†^
Coronary artery aneurysm	3 (7.6)	1 (6)	2 (9)	0.81
Pericarditis/pericardial effusion	19 (49)	7 (41)	12 (54)	0.40
**Radiographic results**				
Pleural effusion	10 (26)	2 (12)	8 (36)	0.16

Data presented as median [P5%, 75%], N (column %), ^††^ data of inflammatory markers in patients: represent number of MIS-C patients with elevated levels to the number of patients tested in each group, *: Satterthwaite *t*-test, ^†^: Wilcoxon rank sum test, ^‡^: Pearson’s chi-square test, ^§^: Fisher’s exact test, LVEF: left ventricular ejection fraction; NT-proBNP: N-terminal pro-B-type natriuretic peptide.

**Table 4:** Management of mild and moderately ill hospitalized MIS-C patients treated with IVIG monotherapy versus combined IVIG and corticosteroid therapy.

Characteristic	IVIG Treatment (n = 17)	IVIG and Corticosteroid Treatment (n = 22)	*p* Value
**General features**			
Duration of symptoms before initiation of therapy (days)	6 [4.5, 6.5]	5 [4, 5]	0.64 *
Time lag between admission and start of therapy (days)	1.0 [0, 1.0]	0.50 [0, 2.0]	0.88 ^†^
**Supportive Care**			
Fluid resuscitation	4 (24)	12 (55)	0.051
Fluid resuscitation before therapy (mL/kg)	0 [0, 10]	10 [0, 23]	0.064 ^†^
Fluid resuscitation after starting therapy (mL/kg)	0 [0, 0]	0 [0, 0]	0.99 ^†^
Vasoactive medications	1 (6)	6 (27)	0.19 ^§^
Nasal cannula oxygen	1 (5.9)	8 (36)	0.052 ^§^
Non-invasive ventilation	1 (5.9)	2 (9.1)	0.99 ^§^
**Medications**			
IVIG			
IVIG dose (gms/kg)	2.0 [2.0, 2.0]	2.0 [2.0, 2.0]	0.99 ^†^
IVIG duration (days)	1.0 [1.0, 1.0]	1.0 [1.0, 1.0]	0.88 ^†^
Corticosteroids			
Steroid dose			0.49 ^§^
Low dose (2 mg/kg)	6 (86)	15 (68)	
High dose (10 mg/kg)	1 (14)	7 (32)	
Steroid dose distribution			0.99 ^§^
Single	2 (28)	7 (32)	
Divided	5 (62)	15 (68)	
Immunomodulation			
IL-1 inhibitor therapy	2 (18)	5 (24)	0.99 ^§^
IL-1 inhibitor dose (mg/kg/day)	2.3 ± 0.35	2.9 ± 1.1	0.29 *
IL-1 inhibitor therapy duration (days)	4.5 ± 0.71	6.2 ± 2.8	0.26 *
Additional therapy (steroids and immunomodulators)	9 (53)	5 (24)	0.051 ^§^
Additional steroids after 24 h	7 (41)	0 (0)	0.001 ^§^
Other medications			
Antiplatelet therapy	14 (82)	20 (91)	0.64 ^§^
Anticoagulation therapy	5 (29)	18 (82)	<0.001 ^‡^

Data presented as mean ± SD, Median [P25, P75], N (column %). *p*-values: *: Satterthwaite *t*-test, ^†^: Wilcoxon rank sum test, ^‡^: Pearson’s chi-square test, ^§^: Fisher’s exact test.

**Table 5:** Cardiovascular parameters and clinical outcomes of mild and moderately ill hospitalized MIS-C patients treated with IVIG monotherapy versus combined IVIG and corticosteroid therapy.

Characteristic	IVIG Treatment (n = 17)	IVIG and Corticosteroid Treatment (n = 22)	95% CI [Lower Limit, Upper Limit]	*p* Value
**Cardiovascular features**				
Delta ↓ in HR (rate/min)				
Day 0 to day 1	11 [3, 19]	32 [27, 41]	17.8 [9.74, 25.8]	<0.001 *
Day 0 to day 3	25 [6.5, 41.5]	44.5 [31, 61]	21.3 [6.6, 36.1]	0.009 *
Day 0 to day 5	37 [28, 55]	64 [45, 75]	22.3 [8.7, 35.9]	0.032 *
Delta ↑ in mean BP (mmHg)				
Day 0 to day 1	5 [1, 7]	8.5 [6, 13]	5.63 [1.61, 9.64]	0.007 *
Day 0 to day 3	7 [3, 16.5]	12 [5, 19]	3.42 [0.8, 10.67]	0.049 *
Day 0 to day 5	9.5 [2.5, 19]	17 [12, 22]	3.71 [−2.5, 9.92]	0.13 *
**General features**				
Fever resolution (days)	2.0 [0, 3.0]	0 [0, 0]	1.48 [0.79, 2.16]	<0.001 *
Delta ↓ in temperature (°C)				
Day 0 to day 1	0.9 [0.25, 1.3]	2.3 [1.5, 2.9]	1.45 [0.94, 1.95]	<0.001 *
Day 0 to day 3	1.9 [1.3, 2.5]	2.6 [2, 3.1]	0.74 [0.21, 1.26]	0.005
**Clinical outcomes:**				
PICU admission following initiation of therapy	5 (29)	5 (22)		0.55 ^†^
PICU length of stay (days) following initiation of therapy	2 [0.5, 7]	4 [2.5, 5.5]	0.01 [−3.21, 3.23]	0.74 *
Hospital length of stay (days)	6 [4, 7]	7.5 [5, 11]	1.66 [−0.44, 3.76]	0.079 *

Data presented as median [P5%, 75%], N (column %), *: Wilcoxon rank sum test, ^†^: Pearson’s chi-square test, day 0 = prior to initiation of therapy, day 1 = 24 h after initiation of therapy, day 3 = 72 h after initiation of therapy, day 5 = 120 h after initiation of therapy; Delta: difference, ↑: increase, ↓: decrease; CI: confidence interval; HR: heart rate; BP: blood pressure; PICU: pediatric intensive care unit.

**Table 6:** Inflammatory markers responses of mild and moderately ill hospitalized MIS-C patients treated with IVIG monotherapy versus combined IVIG and corticosteroid therapy.

Characteristic	IVIG Treatment (n = 17)	IVIG and Corticosteroid Treatment (n = 22)	95% CI [Lower Limit, Upper Limit]	*p* Value
**Inflammatory markers**				
Delta ↑ in albumin levels				
Day 0 to day 1	−0.4 [−0.85, −0.1]	−0.25 [−0.8, 0]	0.01 [−0.34, 0.36]	0.80 *
Day 0 to day 3	−0.2 [−0.9, 0]	0.15 [−0.5, 0.4]	0.43 [0.2, 0.84]	0.035 *
Day 0 to day 5	0 [−0.5, 0.25]	0.4 [−0.3, 0.75]	0.37 [0.1, 0.70]	0.037 *
Delta ↓ in CRP levels				
Day 0 to day 1	1.9 [−0.45, 4.4]	3.6 [1.4, 6.8]	2.32 [−0.35, 4.99]	0.14 *
Day 0 to day 3	4.6 [−0.75, 13]	10 [8.2, 18]	7.56 [3.0, 12.11]	0.019 *
Day 0 to day 5	5.1 [2.9, 14]	13 [9.8, 21]	4.6 [0.2, 9.4]	0.03 *
Delta ↓ in D Dimer levels				
Day 0 to day 1	30 [−220, 1060]	1010 [270, 1710]	985 [−374.1, 2344.6]	0.053 *
Day 0 to day 3	395 [−355, 1345]	1840 [655, 3990]	2344 [488.7, 4200.2]	0.015 *
Day 0 to day 5	630 [−30, 2080]	2900 [1270, 4160]	2486 [854.6, 4117.3]	0.048 *
Delta ↓ in ferritin levels				
Day 0 to day 1	−13 [−56, 60]	31 [−16, 210]	416 [−186, 1019.9]	0.16 *
Day 0 to day 3	12 [−33, 162]	137 [54, 257]	1448 [−609.4, 3505.5]	0.049 *
Day 0 to day 5	128 [1.5, 255]	296 [93, 330]	2350 [−284.1, 4948.1]	0.35 *
Delta ↓ in fibrinogen levels				
Day 0 to day 1	32 [−16, 154]	55 [32, 120]	13.4 [−40.1, 16.8]	0.47 *
Day 0 to day 3	66 [−16, 206.5]	184 [141, 271]	110 [44.4, 176]	0.027 *
Day 0 to day 5	291 [190, 293]	284 [223, 320]	72.3 [−3.4, 147.9]	0.52 *
Delta ↑ in lymphocyte count				
Day 0 to day 1	110 [−130, 280]	−60 [−340, 680]	101.9 [−466.5, 670.3]	0.83 *
Day 0 to day 3	515 [−45, 1095]	1050 [390, 1890]	1006 [63.5, 1948]	0.048 *
Day 0 to day 5	1150 [400, 3740]	1915 [605, 3420]	715.9 [−428.6, 1860]	0.65 *
Delta ↓ in neutrophil count				
Day 0 to day 1	1700 [−230, 2460]	125 [−2180, 2600]	531 [−1819, 2882]	0.52 *
Day 0 to day 3	−40 [−2475, 2430]	−1375 [−4660, 160]	939 [−1717, 3596]	0.23 *
Day 0 to day 5	−940 [−6340, 2225]	−3295 [−5450, 1220]	787 [−2657, 4231]	0.61 *
Delta ↓ in NT - proBNP levels				
Day 0 to day 1	−23 [−391, 182]	597 [−84, 1842]	58.6 [−2104.7, 2222]	0.20 *
Day 0 to day 3	411 [103, 719]	1660 [916, 3640]	2901 [−349.3, 6153]	0.020 *
Day 0 to day 5	697 [66, 1193]	1999 [1032, 5285]	3113 [−851.4 7078]	0.048 *
**Echocardiographic results**				
Delta ↑ in LVEF %				
Day 0 to day 4–5	3 [1, 9.5]	8 [5, 12]	3.84 [0.55, 8.23]	0.032 *

Data presented as median [P25, P75]. *p*-values: * = Wilcoxon rank sum test. Day 0 = prior to initiation of therapy, day 1 = 24 h after initiation of therapy, day 3 = 72 h after initiation of therapy, day 5 = 120 h after initiation of therapy; Delta: absolute difference in test result value, ↑: increase, ↓: decrease; CI: confidence interval; LVEF: Left ventricular ejection fraction; NT -proBNP: N-terminal pro-B-type natriuretic peptide.

## Data Availability

The data for this study shall be shared by the corresponding author upon reasonable request.

## Supplementary Materials

The following supporting information can be downloaded at: https://www.mdpi.com/article/10.3390/jcdd12090324/s1. Table S1: Multi-organ system results of mild and moderately ill MIS-C patients treated with IVIG versus IVIG and corticosteroids admitted to the hospital.

## Abbreviations

CDC	Centers for Disease Control and Prevention
CRP	C Reactive Protein
CTSE	Council of State and Territorial Epidemiologists
GC	Glucocorticoids
IVIG	Intravenous immunoglobulins
LVEF	Left Ventricular Ejection Fraction
MIS-C	Multisystem inflammatory syndrome in children
NT-proBNP	N-Terminal pro Brain Natriuretic Peptide
PICU	Pediatric intensive care unit
SARS-CoV-2	Severe acute respiratory syndrome coronavirus 2
VIS	Vasoactive-Inotrope Score
