# Supplementary material for: Comparison of Immunomodulatory Therapies for Cardiovascular Clinical and Inflammatory Markers Outcomes in Mild to Moderately Ill Hospitalized Multisystem Inflammatory Syndrome in Children Patients
Source: J Cardiovasc Dev Dis. 2025 Aug 25;12(9):324. doi: 10.3390/jcdd12090324 (PMC12471045; doi:10.3390/jcdd12090324)
Supplement: Supplementary file 1 [file jcdd-12-00324-s001.zip › jcdd-3756615-supplementary.pdf]

Supplementary Table S1. Multi-organ system results of mild and moderately ill MIS-C patients treated with IVIG versus IVIG and corticosteroids admitted to the hospital.

Characteristic	All patients (n = 39)	IVIG treatment (n = 17)	IVIG & corticosteroid treatment (n = 22)	P value
**Gastrointestinal**				
Serum bilirubin (mg/dL)	0.5 [0.3, 0.7]	0.4 [0.3, 0.74]	0.45 [0.2, 0.6]	0.222*
Aspartate aminotransferase (U/L)	39 [24, 60]	28 [19, 60]	37 [27, 64]	0.413*
Alanine transaminase (U/L)	30 [17,44]	35 [19, 47]	29 [16, 43]	0.795*
**Renal**				
Serum blood urea nitrogen (mg/dL)	11 [9,15]	10 [8, 16]	12.5 [11, 15]	0.618*
Serum creatinine (mg/dL)	0.5 [0.39, 0.7]	0.51 [0.36, 0.71]	0.49 [0.41, 0.67]	0.606*
Serum sodium (mEq/L)	133 [132, 136]	134 [131, 135]	133 [132, 136]	0.738*
Urinalysis				
Specific gravity	1021 [1013, 1030]	1017 [1012, 1028]	1024 [1013, 1030]	0.516*
Sterile pyuria	9 (23)	4 (24)	5 (23)	0.745†
Hematuria	3 (7)	2 (11)	1 (5)	0.815†
Proteinuria	9 (23)	3 (18)	6 (27)	0.745†
**Pulmonary**				
Chest x-ray				
Infiltrate	1 (2)	0	1 (2)	
Pleural effusion	10 (26)	2 (12)	8 (36)	0.16†
Pneumothorax	0	0	0	

Data presented as Median [P5%, 75%], N (column %), *: Wilcoxon Rank Sum test, †: Pearson's chi-square test
